# Functionally different PIN proteins control auxin flux during bulbil development in *Agave tequilana*

**DOI:** 10.1093/jxb/erv191

**Published:** 2015-04-23

**Authors:** María Jazmín Abraham Juárez, Rocío Hernández Cárdenas, José Natzul Santoyo Villa, Devin O’Connor, Aaron Sluis, Sarah Hake, José Ordaz-Ortiz, Leon Terry, June Simpson

**Affiliations:** ^1^Department of Plant Genetic Engineering, Cinvestav Irapuato, Km. 9.6 Libramiento Norte Carretera Irapuato-León, Apdo. Postal 629, 36821 Irapuato, Guanajuato, Mexico; ^2^Sainsbury Laboratory, Cambridge University, 47 Bateman Street, Cambridge CB2 1LR, UK; ^3^Plant Gene Expression Center, US Department of Agriculture-Agricultural Research Service, Plant and Microbial Biology Department, University of California at Berkeley, Berkeley, CA 94720, USA; ^4^Plant Science Laboratory, Cranfield University, Bedfordshire MK43 0AL, UK

**Keywords:** *Agave tequilana*, auxin flux, bulbil, development, gene expression, immunolocalization, PIN proteins.

## Abstract

In *Agave tequilana*, reproductive failure or inadequate flower development stimulates the formation of vegetative bulbils at the bracteoles, ensuring survival in a hostile environment. Little is known about the signals that trigger this probably unique phenomenon in agave species. Here we report that auxin plays a central role in bulbil development and show that the localization of PIN1-related proteins is consistent with altered auxin transport during this process. Analysis of agave transcriptome data led to the identification of the *A. tequilana* orthologue of PIN1 (denoted AtqPIN1) and a second closely related gene from a distinct clade reported as ‘Sister of PIN1’ (denoted AtqSoPIN1). Quantitative real-time reverse transcription–PCR (RT-qPCR) analysis showed different patterns of expression for each gene during bulbil formation, and heterologous expression of the *A. tequilana* PIN1 and SoPIN1 genes in *Arabidopsis thaliana* confirmed functional differences between these genes. Although no free auxin was detected in induced pedicel samples, changes in the levels of auxin precursors were observed. Taken as a whole, the data support the model that AtqPIN1 and AtqSoPIN1 have co-ordinated but distinct functions in relation to auxin transport during the initial stages of bulbil formation.

## Highlight

Survival of *A. tequilana*, under arid conditions depends on a failsafe mechanism for asexual reproduction based on changes in auxin mobility controlled by two functionally distinct PIN1-related proteins.

## Introduction

Agaves are perennial monocarpic plants with life cycles ranging from 5 to 7 years for *Agave tequilana* (Valenzuela, 2003, 2011) to an estimated 53 years for *A. deserti* (Nobel, 1976), during which carbohydrates accumulate in stem tissue in the form of oligofructans (Lopez *et al.*, 2003; Mancilla-Margalli and Lopez, 2006) which are exploited in *A. tequilana* to produce tequila under a controlled denomination of origin and in other species to produce mescal. Currently much interest has been shown in the potential of agave plants as sources for biofuel production since they are not exploited as food sources and can be grown on land not suitable for production of many traditional crop plants (Borland *et al.*, 2009).

Many species of the *Agave* genus have evolved to survive under arid conditions (Nobel, 1976; Gentry, 1982; Arizaga et al., 2000a, b), and an important adaptation is a failsafe strategy involving the production of thousands of aerial vegetative plantlets or bulbils on the inflorescence (Arizaga and Ezcurra, 1995, 2002). This strategy comes into play when sexual reproduction is inefficient or unsuccessful, and ensures asexual reproduction even in the absence of other flowering individuals and pollinators or in the event of adverse environmental conditions (Arizaga and Ezcurra, 1995, 2002).

The formation of aerial bulbils has been documented in several groups of plant species; however, the mechanisms for inducing and carrying out bulbil development in different species are very distinct. In *Titanotrichum oldhamii*, vegetative meristems develop in the vicinity of the floral meristem when this tissue apparently loses its identity, leading to the formation of vegetative bulbils within the original floral structure (Wang and Cronk, 2003). In *Kalanchoë* species such as *K. diagremontiana*, a somatic embryogenesis programme similar to zygotic embryogenesis is undergone where the transcription factor Leafy Cotyledon1 plays an essential role (Garces *et al.*, 2014).

Bulbil formation is also common in other members of the Liliopsida class (which includes the *Agave* genus), such as *Allium sativum* (Ebi *et al.*, 2000; Mathew *et al.*, 2005) and *Dioscorea japonica* where miniature bulbs are formed in leaf axils or on flower heads. These bulbils are often dormant and will germinate the following season (Kim *et al.*, 2005; Walck *et al.*, 2010). In contrast, agave species directly produce numerous small plantlets at the bracteoles of the inflorescence that eventually fall to the ground and grow independently (Abraham-Juarez *et al.*, 2010). The formation of new meristems following bulbil induction implies that cells in the pedicel tissue are reprogrammed actively to undergo cell division to form vegetative structures similar to the activation of dormant axillary meristems following removal of the shoot apical meristem (SAM) (Leyser, 2005). Thousands of bulbils are produced on a single inflorescence in a few months in comparison with 1–2 offsets produced from the rhizomes of the mother plant each year, and reports have shown a slightly higher level of somaclonal variation in bulbils in comparison with offsets (Gonzalez *et al.*, 2003; Diaz-Martinez *et al.*, 2012).

A better understanding of the process of bulbil development will provide valuable information regarding the developmental and molecular mechanisms associated with this process in comparison with that of pre-formed, dormant axillary meristem development in other species where the number of meristems and new branches is strictly controlled. The plant hormone auxin plays an essential role in orchestrating the developmental changes that occur throughout a plant’s life cycle (Bohn-Courseau, 2010; Peer, 2013) and, when auxin gradients are altered, developmental and growth patterns are also modified (Mai *et al.*, 2011; Goh *et al.*, 2012; Rademacher *et al.*, 2012). In particular, removal of the SAM leads to activation of dormant axillary meristems by a mechanism thought to involve localized changes in the balance of auxin and cytokinin levels (Muller and Leyser, 2011). Expression of several transcription factors is also affected by auxin including the KNOX, MADS, and LEAFY genes which have previously been implicated in bulbil induction in *A. tequilana* or *T. oldhamii* (Wang *et al.*, 2004; Abraham-Juarez *et al.*, 2010; Delgado Sandoval *et al.*, 2012). These observations suggest that the modulation of auxin gradients following removal of flower buds in *A. tequilana* might also determine the initiation of bulbil formation.

The synthesis, degradation, conjugation, or transport of auxin molecules regulates the presence of auxin within specific plant tissues. In particular, auxin mobility mediated by polarized auxin efflux transporters known as PIN-formed (PIN) proteins plays an important role in determining developmental responses to environmental or physical cues. PIN proteins are composed of two highly conserved transmembrane domains that flank a more variable hydrophyllic loop. PIN proteins with long loop domains are located at the cell membrane in a polarized fashion where they mediate the directional flow of auxin within plant tissues. A detailed analysis of PIN1-related proteins from grass species identified a new clade ‘Sister of PIN1’ (SoPIN1) and, based on inmunolocalization and computer simulation, a model for the distinct roles of PIN1 and SoPIN1 during organ initiation was proposed (O’Connor *et al.*, 2014). In addition, different combinations of motifs within the hydrophilic loop region are consistent with the functional divergence of the PIN proteins (Wang *et al.*, 2014).

The aim of this work was to determine the role of auxin in the stimulation of bulbil development in *A. tequilana* and to explore the role of auxin transport mediated by PIN1-related proteins during this process. The characterization of two distinct *A. tequilana* PIN1 genes from different clades and their contrasting patterns of expression during bulbil development are described. By analysis of PIN1 protein localization, the formation of basipetal auxin gradients during the formation of vegetative propagules in *A. tequilana* is shown when the apical floral meristem is cut, demonstrating overlap between apical dominance and bulbil formation. By means of exogenous application of auxin, the role of auxin in the suppression of bulbil formation is also demonstrated for the first time and, based on the results, a preliminary model for the control of this process at the molecular level is proposed.

## Materials and methods

### Plant material

Bulbil samples were obtained at the same stages of development as described previously (Abraham-Juarez *et al.*, 2010): S0, immediately before bud excision; S1, 7 d after bud removal (no visible change observed); S2, swelling; S3, necrosis; S4, bulbil eruption; and S5, nascent bulbils present (Supplementary Fig. S1A, B available at *JXB* online). These tissues were used to obtain mRNA and perform real-time PCR assays and immunolocalization assays.

### Application of exogenous auxin

Three plants of *A. tequilana* with a 1 m long inflorescence were transferred from commercial fields to a greenhouse. They were monitored until flowering, and, when floral buds were 5cm long, bulbil induction was carried out by excision of the buds. Auxin or control treatments were applied to the pedicel daily beginning immediately after bud excision. Treatments were: (i) lanolin only as a control; (ii) lanolin containing 100 μM indole acetic acid (IAA); and (iii) lanolin containing 100 μM naphthylphthalamic acid (NPA). Treatments were carried out in summer (July–August), with a daylength of 14h light and 10h darkness. Tissue collection and treatment application were carried out in the morning. Each plant was divided into three sections and all treatments were applied to each individual plant. Pedicel tissue was collected for quantitative real-time reverse transcription–PCR (RT-qPCR) analysis and immunolocalization, and stored at –70 °C or fixed in FAA until needed. All samples were collected from the three individual plants as biological replicates.

### Immunolocalization

Tissues were fixed in FAA [50% ethanol, 3.7% formaldehyde, 5% acetic acid, 0.5% Triton X-100, and 1% dimethylsulphoxide (DMSO)] overnight and were then dehydrated through an ethanol series and infiltrated with polyester wax (Steedman, Electron Microscopy Sciences). Following embedding and sectioning, 10 μm sections were placed onto Probe on plus^®^ slides (Fisherbrand^®^). For antibody reactions, polyester wax was removed in 100% ethanol. The sections were then rehydrated in an ethanol series and blocked by incubating in phosphate-buffered saline (PBS) buffer (130mM NaCl and 10mM PO_4_) with 1% bovine serum albumin (BSA). The slides were incubated with an affinity-purified anti-*Zea mays* PIN1a antibody (Hake Lab; 1:200 dilution) in PBS with 1% BSA overnight at 4 °C. After three washes in PBS with 1% fish gelatin, the slides were incubated with a Cy3-conjugated anti-guinea pig secondary antibody (1:100 dilution; Jackson Immuno Research Laboratory). Slides were washed five times in PBS with 1% fish gelatin, mounted in 1% DABCO in 90% phosphate-buffered glycerol, and observed by fluorescence microscopy at 540nm using the same exposure time for all samples. Photographs were taken using an Olympus BX60 microscope fitted with an Olympus DP71 camera.

### Sequence analysis

To identify PIN1 cDNA sequences within the *A. tequilana* transcriptome, two types of search were carried out: by key word and by using the BLAST tool to search specifically for PIN1-domain sequences. A threshold of E=10^–6^ was used to select sequences of interest. Alignments of amino acid sequences were performed using the Clustal W software (Larkin *et al.*, 2007). A phylogenetic tree was constructed using the Neighbor–Joining (NJ) and maximum likelihood method in the MEGA 5.0 software suite (http://www.megasoftware.net/), and robustness was assessed by bootstrap analysis (Felsenstein, 1986) with 1000 replicates. Previously reported *Arabidopsis*, rice, and maize PIN proteins were used to classify the *A. tequilana* PIN sequences and name them accordingly. AtqPIN protein transmembrane helices were predicted using TMHMM2 (Krogh *et al.*, 2001). Amino acid motifs were identified and annotated using Geneious software (http://www.geneious.com). AtqPIN1-1 and AtqSoPIN1 sequences have been deposited in GenBank under accession numbers: BankIt1715396 AtqPIN1 KJ676663 and BankIt1715396 AtqSoPIN1 KJ676664.

### Quantitative real-time reverse transcription–PCR analysis

RT-qPCR was carried out for all tissues sampled. Total RNA was isolated using the PureLink Micro-to-Midi Total RNA Purification System (Ambion^R^) according to the manufacturer’s instructions. cDNA was synthesized using a reverse primer mix consisting of 1 μg of RNA, and RevertAid™ Reverse Transcriptase (Fermentas) on three independent replicates. All samples were run in duplicate, and qPCR was carried out for each cDNA replicate. The specific primers used for RT-qPCR are shown in Supplementary Table S1 at *JXB* online. PCRs were performed under the following conditions: 95 °C for 10min, 40 cycles of 95 °C for 15 s, 60 °C for 1min, and a final melt curve stage from 60 °C to 95 °C. Control reactions were carried out using specific primers for the *ACT2* agave gene. For qPCR, the Step One Plus Real Time PCR System (Applied Biosystems) was used. SYBR Green PCR Master Mix (Applied Biosystems) was used for all reactions according to the manufacturer’s protocol. Gene expression was normalized by subtracting the C_T_ value of the control gene from the C_T_ value of the gene of interest. Average expression ratios were obtained from the equation 2^–ΔΔCT^, where ΔΔC_T_ represents ΔC_T_ (gene of interest in stage evaluated)–ΔC_T_ (gene of interest at T0), according to the protocol reported by Czechowski *et al.* (2004).

### Analysis of auxin, precursors, and conjugates

For quantification of IAA and related metabolites, extraction was carried out as described in Novak *et al.* (2012), using 10mg of dry tissue. To validate the quantification of endogenous auxin metabolites, the following stable isotope-labelled internal standards were added as tracers: [^2^H_5_]IAA, [^2^H_5_]IAAsp, [^2^H_2_]TRA, and [^2^H_5_]TRP (100 pmol per sample). Samples were analysed by UHPLC-Q-TOF MS (ultra high-pressure liquid chromatography/quadrupole time-of-flight mass spectrometry; Agilent Technologies, http://www.home.agilent.com), operating in positive ESI (electrospray ionization) mode. A 5 μl aliquot of each sample was injected onto a reversed-phase column [Waters Acquity CSH™ C18 1.7 μm, 2.1×50mm column, eluted using a 20min gradient comprising 0.1% acetic acid in water (A) and 0.1% acetic acid in methanol (B) at a flow rate of 0.2ml min^–1^], column temperature 30 °C. The following binary linear gradient was used: 0min, 90:10 A:B; 10.0min, 50:50 A:B; 12.5min, 40:60 A:B. At the end of the gradient, the column was washed with 100% methanol (2.5min), and re-equilibrated to initial conditions (5min). The eluent was introduced into the Agilent jet stream dual ESI source with the optimal settings as follows: drying gas temperature, 250 °C; drying gas flow, 10 l min^–1^; nebulizer pressure, 60 psi; sheath gas temperature, 400 °C; sheath gas flow, 12 l min^–1^; capillary voltage, 2250V; nozzle voltage, 500V. Peak integration was carried out under full scan conditions by using the extracted ion chromatogram (EIC) of the protonated molecule from the total ion chromatogram (TIC) with a 20 ppm mass extraction window. The accurate mass spectrum and retention time for all compounds were used for both confirmation and quantification purposes, with peak areas of EICs being used for quantification. Chromatograms were analysed using MASSHUNTER software version B.04.00 (Agilent Technologies).

## Results

### Removal of *A. tequilana* flower buds results in the formation of vegetative bulbils and/or new flower buds

**Fig. 1.**
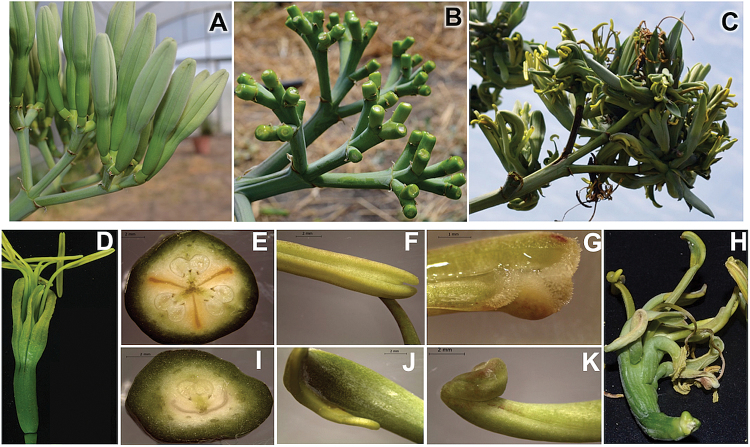
Removal of flower buds leads to formation of vegetative or floral structures at bracteoles in *A. tequilana*. (A) Unopened floral buds. (B) Pedicel tissue following removal of buds. (C) Vegetative bulbils and floral structures formed at bracteoles. (D–G) Normal flower morphology. (D) Completely normal flower; (E) normal carpelar organization shown in cross-section; (F) normal anther; (G) normal pistil. (H–K) Morphology of floral structures formed at bracteoles. (H) Completely distorted flower; (I) distorted carpelar organization; (J) distorted anther; (K) stigma-like structure. Scales are 2.0mm. (This figure is available in colour at *JXB* online.)

In *A. tequilana*, bulbils are naturally produced close to the bracteoles when sexual reproduction fails and no fertilized seeds are produced. These structures can also be induced by removal of flower buds (Fig. 1A, B). Under field conditions, the majority of vegetative bulbils which form after floral bud removal produce viable plantlets that can eventually be removed from the mother plant and grown individually; however, removal of flower buds under greenhouse conditions leads to the formation of a majority of structures morphologically similar to flower buds or with mixed identity, where both vegetative leaves and reproductive organs are present (Fig. 1C). Detailed analysis of flower-like structures showed that all floral tissues including ovaries, anthers, carpels, pistils, and tepals have a distorted morphology (Fig. 1D–K). However, germination *in vitro* of pollen obtained from these secondary flower structures shows similar efficiency (42%) to that obtained from normal flowers (36%), suggesting that the distorted morphology of the maternal organs (ovaries and carpels) could explain why successful fertilization and formation of capsules and seeds is never observed in secondary flower structures. Overall these results show that induction of bulbil formation by removal of flower buds under normal field conditions in general leads to the formation of indeterminate vegetative meristems that produce new plantlets, whereas under greenhouse conditions, possibly due to changes in light intensity, determinate floral meristems are predominantly produced.

Expression patterns of agave *KNOX* and *MADS* box genes during bulbil formation were previously shown to be consistent with the expression patterns expected for vegetative growth; *AtqKNOX1*, *AtqKNOX2*, and *AtqMADS5* transcripts increased and *AtqMADS1* transcription decreased (Abraham-Juarez *et al.*, 2010; Delgado Sandoval *et al.*, 2012). In order to determine whether the expression patterns of these genes were reflected in the type of secondary structure formed (vegetative or floral), the expression patterns of the agave KNOX genes *AtqKNOX1* and *AtqKNOX2* and the agave MADS box genes *AtqMADS1* and *AtqMADS5* were determined during the formation of predominantly floral structures in greenhouse conditions. As shown in Supplementary Figure S2A–D at *JXB* online, the expression patterns correlate with the formation of floral rather than vegetative structures. Both *AtqKNOX1* and *AtqKNOX2* genes and *AtqMADS5* (associated with vegetative growth) show a decrease in expression as determinate floral meristems are formed, whereas *AtqMADS1*, associated with flower development, increases in expression as floral structures develop and decreases to a minimum level of expression in stage S5 when determinate floral structures are observed.

### Auxin as a major regulator of the cellular switch in bulbil formation in *A. tequilana*

**Fig. 2.**
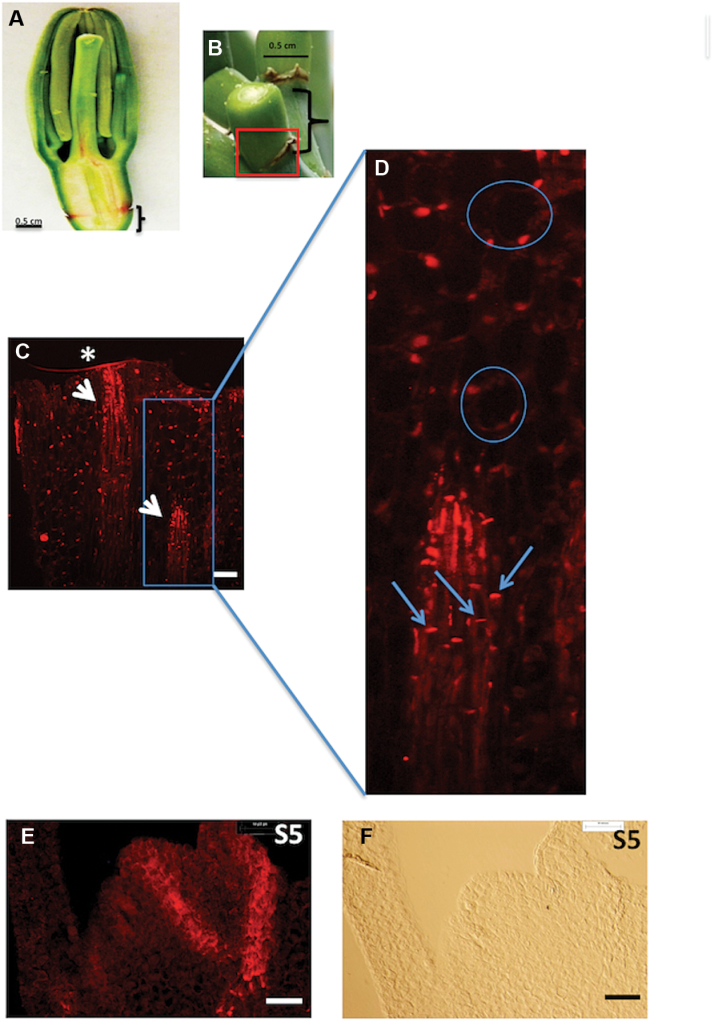
Immunolocalization of agave PIN protein in pedicel and S5 bulbil tissue. (A) Bisected bud before sectioning. (B) Example of pedicel tissue with floral bud removed; the box indicates the region surrounding the bracteoles on which immunolocalization of PIN was carried out; brackets indicate pedicel tissue. (C) Immunolocalization of PIN protein using an anti-PIN1a antibody against maize PIN1a protein, in pedicel tissue where bud has been removed as shown in (B). An asterisk (*) indicates the cut surface. (D) Enlarged image showing PIN location in the lower periphery of cells; circles indicate individual cells outside vascular tissue; arrows indicate PIN localization in individual cells within the vascular tissue. Scale bar in (C) is 100 μm. (E) Light microscopy and (F) PIN immunolocalization images of untreated pedicel tissue (box in B), underlying the region where a floral bud was removed at the S5 stage of bulbil induction, ×20 magnification, scale bar=50 μm. (This figure is available in colour at *JXB* online.)

It was hypothesized that removal of flower buds or ineffective fertilization in agave leads to modification of auxin levels and/or auxin transport in the underlying pedicel tissue which in turn leads to the induction of new meristems at the bracteoles. Since polar auxin transporters of the PIN family have been reported to play a central role in the formation of auxin gradients during organ formation (Bohn-Courseau, 2010), it was decided to examine patterns of PIN protein localization during bulbil formation. Figure 2a shows an example of a bisected floral bud and the underlying pedicel tissue, and Fig. 2b an example of pedicel tissue from which the bud has been removed. Figure 2c and d shows a light microscopy image and PIN immunolocalization image, respectively, at T0 (immediately after bud removal). PIN protein is observed in vascular tissue and at the lower periphery of both vascular and surrounding cells, suggesting auxin transport basipetally from the flower bud region. When vegetative bulbils begin to form, a localized concentration of PIN in a polar configuration typical of apical meristems is observed at the S5 stage, suggesting that a basipetal flux of auxin from the developing SAMs is established as the new vegetative structures develop (Fig. 2e, f).

**Fig. 3.**
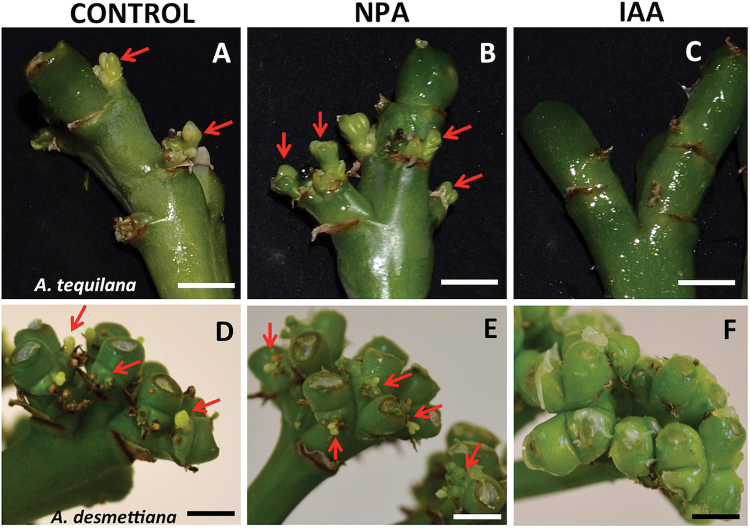
Exogenous application of NPA and IAA to the cut surface of pedicel tissue following removal of floral buds. (A–C) *Agarve tequilana* 10 d after bud removal. (A) Lanolin-only control; (B) NPA; (C) IAA. (D–F) *Agarve desmettiana* 10 d after bud removal. (D) Lanolin-only control; (E) NPA; (F) IAA. Meristems are indicated with arrows. Bars are 0.5cm. (This figure is available in colour at *JXB* online.)

To test the hypothesis that auxin plays a central role in regulating bulbil formation, three different treatments were applied to pedicel tips from which floral buds had been removed for both *A. tequilana* and *A. desmettiana*: (i) exogenous application of IAA; (ii) exogenous application of the auxin transport inhibitor NPA; and (iii) application of a lanolin-only control (see the Materials and methods). As shown in Fig. 3A–F, application of exogenous auxin on cut pedicel tissue suppressed bulbil formation at the bracteoles, whereas the application of NPA or a lanolin-only control led to the formation of new meristems and bulbil structures. Bulbil auxin treatments were repeated in three consecutive years for *A. tequilana* and in two consecutive years for *A. desmettiana.* Similar results were obtained in all cases. These results demonstrate that pedicel tissue is only capable of developing active meristems in the absence of apical auxin, confirming that apical auxin determines the induction of reprogramming of the necessary cellular processes. Exogenous application of NPA did not inhibit the formation of new meristems.

### Identification and classification of *A. tequilana* PIN1-related cDNAs

**Fig. 4.**
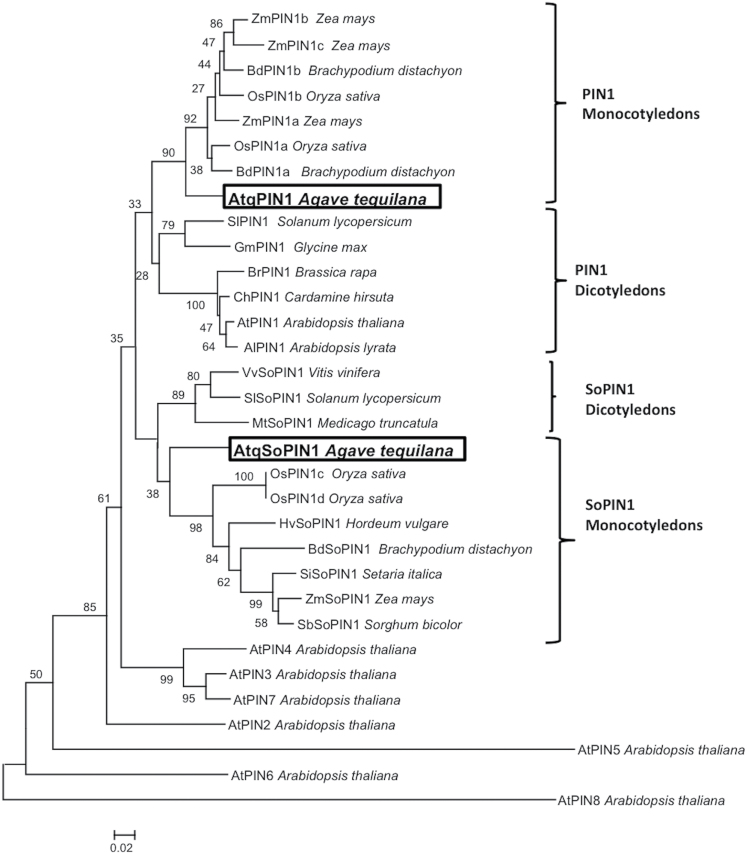
Dendrogram showing the relationship between PIN1 amino acid sequences from monocotyledonous and dicotyledonous species and *A. tequilana*. The agave sequences are boxed. PIN1 and ‘Sister of PIN1’ (SoPIN1) clades containing dicotyledonous or monocotyledonous species are indicated.

Fourteen expressed sequence tags (ESTs) with highly significant homology to PIN1 sequences, most of which were expressed in SAM tissue or bulbil-induced pedicel tissue, were identified in an *A. tequilana* transcriptome database (Avila de Dios and Simpson, unpublished). Alignment of overlapping PIN ESTs produced the complete open reading frames of two putative *A. tequilana* PIN genes (Supplementary Fig. S3 at *JXB* online). Comparison with PIN amino acid sequences from a variety of plant species using both NJ and maximum likelihood methods allowed the identification of two distinct *A. tequilana PIN*-like genes: *AtqPIN1* which is orthologous to other *PIN1* genes and *AtqSoPIN1* which groups within the SoPIN1 clade which is absent in Brassicacea species (O’Connor *et al.*, 2014) as shown in Fig. 4. The percentage identity between the agave protein sequences and ZmPIN1a/ZMSoPIN1 also supports this classification (Supplementary Table S2). AtqPIN1 is 69 amino acids longer than AtqSoPIN1 in the hydrophilic domain, and comparison of the presence of conserved motifs in this region based on the report of Bennett *et al.* (2014) produced a complex pattern. Fifteen different motifs of the 32 motifs analysed (highly variable motifs were not included) were found in each agave PIN protein; however individual motifs differed between AtqPIN1 and AtqSoPIN1. A list of motifs found in the *A. tequilana* PIN1 and SoPIN1 clades, *A. thaliana* PIN1, and *Z. mays* PIN1a and SoPIN1 proteins is given in Supplementary Table S3. Motifs S1, S2, HC2-4, HC2-1-1, and S11 are found in the PIN1 clade proteins but not in the SoPIN1 clade, whereas S7 and S20 are found only in the SoPIN1 clade but not the PIN1 clade. The distribution of these motifs is shown in Supplementary Fig. S4. The motifs denoted HC are found within highly conserved regions of the hydrophilic loop of the proteins analysed. Only *A. thaliana* PIN1 and AtqSoPIN1 have the HC1-4 motif. Differences within these HC motifs are candidates to determine the particular function and localization of the distinct classes of PIN proteins.

### Expression of agave *PIN1*-related genes during bulbil formation

**Fig. 5.**
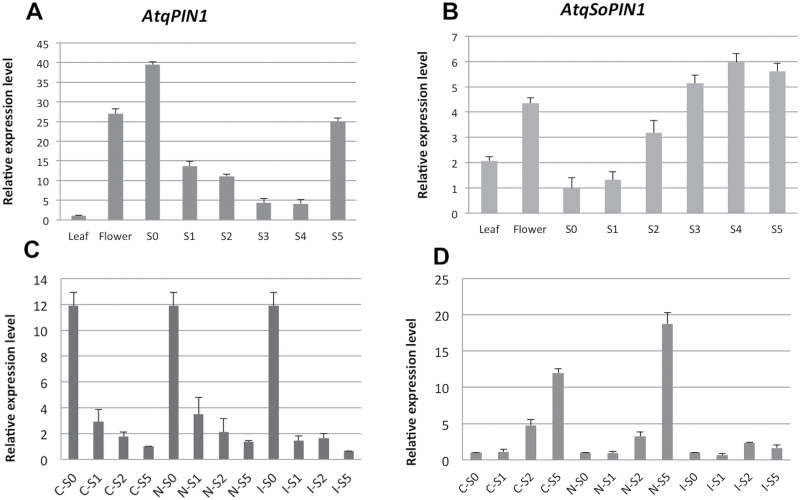
RT-qPCR expression profiles of agave *PIN1* genes in pedicel tissue at different stages of bulbil formation in the presence or absence of NPA or IAA. (A and C) *AtqPIN1*. (B and D) *AtqSoPIN1*. S0–S5, stages of bulbil formation; C-, application of lanolin-only control; N-, application of NPA; I-, application of IAA.

To determine the patterns of expression of *AtqPIN1* and *AtqSoPIN1* during bulbil formation, RT-qPCR was carried out throughout the different stages of bulbil development. As shown in Fig. 5A and B, *AtqPIN1* and *AtqSoPIN1* showed opposing patterns of expression from S0 to S4 stages. *AtqPIN1* showed the highest level of expression in non-induced tissue (S0 stage) followed by a decrease 7 d after induction (S1 stage). Expression continued to decrease in S2 and S3 stages, reaching the lowest level in the S4 stage (28 d after induction). An increase was observed at the S5 (35 d after induction) stage when auxin gradients at bulbil SAMs are established (Fig. 5A). This result is in agreement with results from the immunolocalization analysis where PIN protein is observed when vegetative meristems are formed (Fig. 2e, f).

In contrast, *AtqSoPIN1* showed a lower level of expression in non-induced pedicel tissue (S0) in comparison with leaf tissue and increased gradually in stages S1, S2, and S3, to reach a maximum in S4 before decreasing slightly in S5 (Fig. 5B). These contrasting patterns of expression of the *A. tequilana PIN1* genes suggest that they could be differentially regulated by auxin and play different functional roles.

Analysis of *A. tequilana PIN1* gene expression in pedicel tissue to which exogenous IAA, NPA, or lanolin alone had been applied to the cut surfaces where flower buds had been removed confirmed the differential patterns of expression of *AtqPIN1* and *AtqSoPIN1* during bulbil induction (Fig. 5C, D). Interestingly, the application of exogenous auxin, NPA, or lanolin alone did not affect the patterns of expression of *AtqPIN1* in stages S1–S2. *AtqPIN1* was expected to increase in the S5 stage in control and NPA-treated samples; however, the lack of increase observed may be due to the development of both vegetative and floral structures in the plants analysed in Fig. 5C and D, whereas very few or no floral structures were observed in the samples that were used for analysis in Fig. 5A and B. In contrast, application of exogenous auxin strongly affected the pattern of *AtqSoPIN1* expression in pedicel tissue. An increase in expression was observed in untreated (Fig. 5B), lanolin-only, and NPA-treated samples (Fig. 5D, C-S0 to C-S5 and N-S0 to N-S5); however, no change in expression was detected with the addition of auxin, suggesting that auxin inhibits expression of *AtqSoPIN1*.

### Analysis of the components of auxin metabolism in pedicel tissue during bulbil formation

**Fig. 6.**
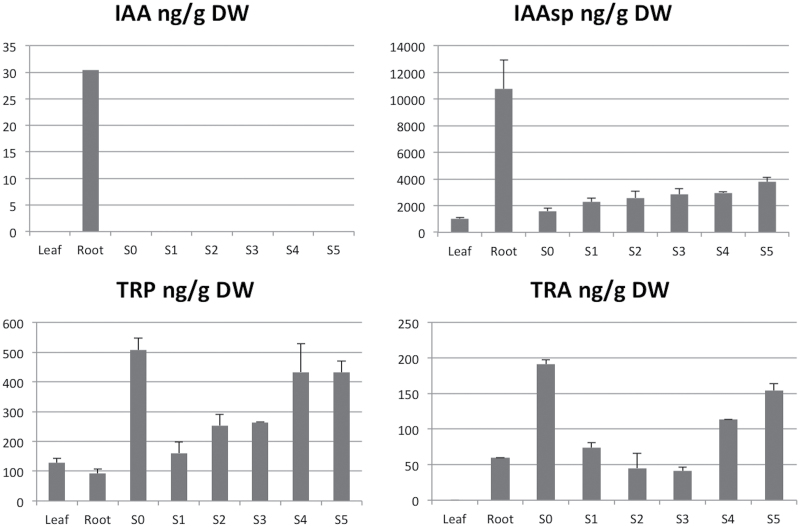
Quantification of free and conjugated forms of IAA and IAA precursors (TRP and TRA) by UHPLC-Q-TOF MS in different stages of bulbil formation in *A. tequilana*. S0–S5: different stages of bulbil formation; DW, dry weight. Leaf and root samples are included as controls.

To determine the role of auxin synthesis and turnover during bulbil development, the levels of free auxin (IAA), a conjugated form of auxin (IAAsp), and the auxin precursors TRP (tryptophan) and TRA (tryptamine) were determined by UHPLC-Q-TOF MS (calibration parameters are shown in Supplementary Table S4 at *JXB* online). Tissue analysed included pedicel tissue in the different stages of bulbil development, and leaf and root tissue as controls. No free IAA was detected in pedicel tissue at any of the stages of bulbil development (Fig. 6). Free IAA was only detected in root tissue. The auxin precursors TRP and TRA were detected in roots and all pedicel tissues tested. TRP but not TRA was also detected in leaf tissue, and varying concentrations of both molecules were observed during bulbil development, with the highest concentrations observed in non-induced pedicel tissue (S0). On bulbil induction, the levels of both precursors decreased ~5- and 2.6-fold for TRP and TRA, respectively. TRP levels increased from stage S1 until S4, and remained at the S4 level in the S5 stage. In contrast, levels of TRA continued to decrease until S3 to an ~5-fold lower level than in non-induced samples at S3, and then increased ~3.7-fold until stage S5. The auxin conjugate IAAsp was also detected in all tissues, with the highest concentration found in root tissue. A low level of IAAsp was observed in non-induced pedicel tissue and slowly increased through S1–S5 to reach an almost 2-fold higher concentration in S5.

### Heterologous expression of agave PIN genes in *Arabidopsis thaliana*

**Fig. 7.**
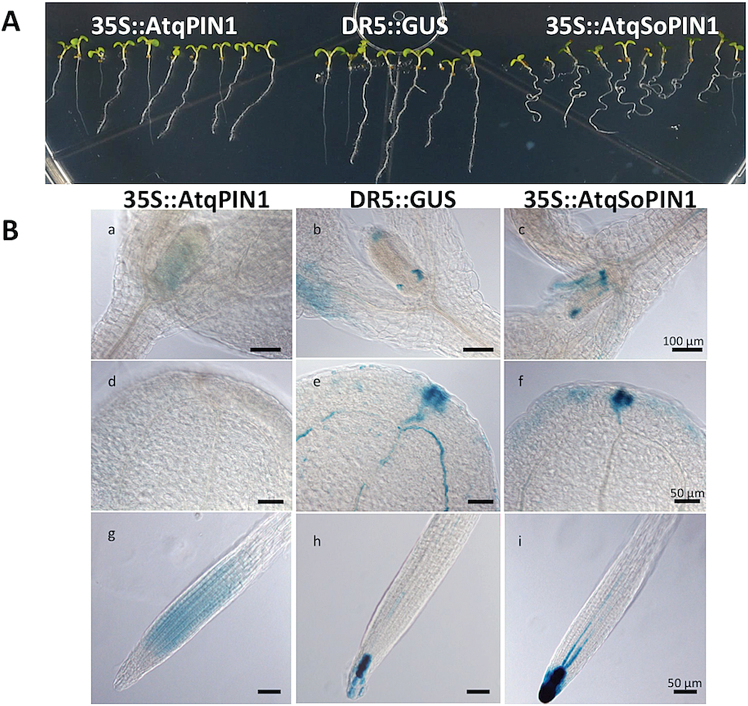
Heterologous expression of *A. tequilana PIN1* and *SoPIN1* genes *in A. thaliana*. (A) Gravitropic responses of DR5::GUS *A. thaliana* seedlings expressing *AtqPIN1* or *AtqSoPIN1* under control of the 35S promoter and DR5::GUS control. (B) Auxin localization based on GUS-stained tissues of DR5::GUS *A. thaliana* plants expressing *AtqPIN1* or *AtqSoPIN1* under control of the 35S promoter and DR5::GUS control. (This figure is available in colour at *JXB* online.)

To examine the function of the *AtqPIN* genes, DR5::GUS (β-glucuronidase) *Arabidopsis* lines that express *AtqPIN1* and *AtqSoPIN1* under control of the 35S promoter were produced. Ectopic expression of *AtqSoPIN1* showed an altered gravitropic response in comparison with the DR5::GUS control line (Fig. 7A), whereas expression of *AtqPIN1* in *A. thaliana* had no effect on gravitropism but showed slightly shorter roots in comparison with the DR5::GUS line. The pattern of auxin response shown by GUS staining was similar in *AtqSoPIN1* and control DR5::GUS lines in the aerial parts of the plant (Fig. 7B b, c, e, f), but in *AtqPIN1*-expressing lines points of auxin accumulation were more diffuse or absent (Fig. 7B a, d, g). In root tissue, significant differences in patterns of auxin localization were observed. *AtqPIN1* lines showed a diffuse pattern of auxin distribution in the region above the quiescent centre and meristematic region, whereas in *AtqSoPIN1*-expressing plants auxin is strongly concentrated in the root tip both at and below the quiescent centre in comparison with the DR5::GUS control which shows auxin localization in cells very close to the quiescent centre (Fig. 7B g–i). These data suggest that, although closely related, AtqPIN1 proteins are functionally distinct at least in the heterologous system of *A. thaliana.*

## Discussion

Many species of agave such as *A. tequilana* produce bulbils when sexual reproduction is unsuccessful, and this may occur throughout the inflorescence or on particular umbels. Other species such as *A. desmettiana* appear to flower normally but are naturally infertile and only produce bulbils rather than seeds. Under field conditions, bulbil induction in both species results in development of predominantly indeterminate, vegetative meristems from which viable independent plantlets are formed. However, plants induced to form bulbils under greenhouse conditions produced predominantly non-viable propagules with a mixture of floral and vegetative organs. The floral organs produced under greenhouse conditions are invariably misshapen and non-viable in reproductive terms. These results confirm the report of Abraham-Juarez *et al.* (2010), which described the formation of new meristems in the bracteolar regions of induced pedicel tissue; however, they also demonstrate that the identity of the meristems formed (determinate or indeterminate) depends on environmental factors. Since temperature, substrate, and manipulation were essentially the same for both garden- and greenhouse-grown plants, changes in light quality or intensity are most probably responsible for the differences observed in bulbil morphology. Interestingly, a recent report described the importance of red light for the efficient production of bulbils in *Pinellia ternata* (Chen *et al.*, 2013).

Contrasting expression patterns of the *KNOX* and *MADS* box genes support the change in meristem identity under different conditions. Light has been shown to have an effect in STM KNOX1 gene expression, as reported by Soucek *et al.* (2007), where activation of isopentenyl transferase repressed STM especially at low light. These findings suggest that high levels of cytokinin repress KNOX1 genes in plants grown in low light. However, the formation of distorted and non-viable floral organs suggests that during the development of the secondary structures, meristem identity was not clearly defined, leading to a mixture of vegetative and floral organs, although ultimately a determinate meristem was produced.

Exogenous application of auxin to the cut surfaces in the absence of the flower bud suppresses bulbil formation, whereas removal of the auxin source allows new vegetative structures to form. Immunolocalization experiments showed localization of PIN proteins with the flux in normal pedicel tissue with intact flowers and in induced pedicel tissue in the latter stages of bulbil induction. Since the PIN1a antibody was raised to a large region of the maize PIN1a protein, with partial homology between AtqSoPIN1 and AtqPIN1 (Supplementary Fig. S3 at *JXB* online), it cannot be determined precisely which proteins were detected in *A. tequilana* tissue. However, based on the model of O’Connor *et al.* (2014), the observation that the PIN1 proteins are oriented with the flux suggests that the observed protein is probably AtqPIN1.

The search for PIN1 ESTs in the *A. tequilana* transcriptome database identified an *AtqPIN1* gene related to maize *ZmPIN1a*, *b*, *c* genes and an *AtqSoPIN1* gene related to members of a recently described new PIN clade *SoPIN1* (O’Connor *et al.*, 2014) which is absent in the Brassicacea. Both *AtqPIN1* and *AtqSoPIN1* transcripts were expressed throughout the different stages of bulbil formation but showed contrasting patterns of expression. Whereas *AtqPIN1* transcripts decreased in response to flower bud excision both during bulbil induction in untreated pedicels and after auxin treatment, *AtqSoPIN1* expression increased with bulbil induction, and this response was suppressed with auxin treatment. These results suggest that the apical tissues are necessary to maintain *AtqPIN1* expression; however, application of exogenous auxin does not maintain *AtqPIN1* expression although bulbil formation is inhibited, but does suppress *AtqSoPIN1* expression. A more detailed study of *PsPIN1* expression in decapitated and auxin-treated stem stumps of pea (Balla *et al.*, 2011) revealed that after decapitation and auxin treatment the *PsPIN1* expression increased 5-fold during the first 12hours but after 5 days dropped below the level observed in intact plants although axillary bud formation remained inhibited. Unchanged expression of *AtqSoPIN1* after auxin treatment is especially interesting since it is in accordance with the observation of O’Connor *et al.* (2014) where *BdSoPIN1* is highly expressed in the epidermis and localized at the points of lemma initiation. This process is similar to the formation of auxin maxima during the initial stages of bulbil development, therefore no change in *AtpSoPIN1* would be expected if bulbil formation is inhibited.

Although removal of floral buds leads to an initial decrease in *AtqPIN1* transcript levels, the pattern of PIN protein immunolocalization in vascular tissue remains unchanged when exogenous auxin is applied. In untreated pedicel samples, at the S5 stage, *AtqPIN1* gene expression increased in correlation with the polarized pattern of immunolocalized PIN protein associated with the formation of new SAMs. However, unexpectedly, no increase in *AtqPIN1* expression was observed in lanolin-only or NPA-treated samples at the S5 stage, although polarized PIN protein was observed at the S5 stage in the typical conformation, indicating a downward flux of auxin from meristem regions.

*AtqSoPIN1* expression remains unchanged when exogenous auxin is applied because no auxin maxima are formed but increases in expression when basipetal auxin transport is interrupted and new meristems are formed. The highest levels of expression in the S5 stage of bulbil development for both lanolin-only and NPA-treated plants support this conclusion. *AtqPIN1* expression decreases in untreated samples, and only in the S5 stage is a downward flux of auxin established, reflected by both *AtqPIN1* expression and AtqPIN1 localization. Since the stages of bulbil formation are determined by morphology and not time after induction, it is unlikely that the lack of increased expression of *AtqPIN1* and AtqPIN1 protein in lanolin-only and NPA-treated samples is due to delayed development where the true S5 stage was reached later in this particular series of experiments. The interpretation of this is therefore that the lack of *AtqPIN1* expression and AtqPIN1 protein is probably due to the presence of predominantly determinate floral meristems in these samples.

Suppression of bulbil formation is maintained in pedicel tissue receiving exogenous auxin, although *AtqPIN1* expression decreases, suggesting that synthesis of new AtqPIN1 protein is not necessary for this process. In contrast, increasing levels of *AtqSoPIN1* expression suggest that newly synthesized SoPIN1 protein could be involved in the auxin flux needed to concentrate this hormone close to the bracteoles where new meristems initiate and develop to form bulbils.

At the amino acid sequence level, AtqPIN1 and AtqSoPIN1 are distinguished by the presence or absence of specific motifs that in general correlate with their classification in distinct clades. One exception, however, is motif HC1-4 within a highly conserved region of the hydrophilic loop that is only strongly supported in AtqSoPIN1 and the *A. thaliana* PIN1 protein. Perhaps unexpectedly, ectopic expression of AtqSoPIN1 in *A. thaliana* produced morphological effects such as altered gravitropism and patterns of auxin localization similar to ectopic expression of the endogenous *A. thaliana* PIN1 gene as reported by Mravec *et al.* (2008), suggesting that AtqSoPIN1 may be functionally more closely related to *A. thaliana* PIN1. In contrast, ectopic expression of the orthologous AtqPIN1 protein that does not carry the HC1-4 motif in *A. thaliana* showed a very different pattern of auxin localization in both aerial and root tissue, and no altered gravitropism, suggesting that this motif may contribute to the functional differences between the agave PIN proteins observed in *A. thaliana*. Because of the absence of SoPIN1 in Brassicacea, the PIN1 protein in these species must be able to act in pumping auxin both against the gradient, thus forming auxin maxima, and also with the flux, draining excess auxin from this maxima. The similar auxin distribution pattern of ectopic expression of *AtqSoPIN1* and *AtPIN1* in A. thaliana with strong auxin maxima could suggest that the HC1-4 motif absent in AtqPIN1 plays an important role in the ability for auxin maxima formation.

PIN1 and SoPIN1 clades may have overlapping functions depending on the specific motifs present in each protein in different species, and perhaps this is how Brassicacea family plants were able to deal with the loss of SoPIN1. It will be interesting in future studies to analyse in detail the distribution of PIN1 and SoPIN1 proteins and the expression patterns of AtqPIN1/SoPIN1 in *A. tequilana* roots.

The lack of motif 11 in the SoPIN1 proteins is also interesting since phosphorylation of conserved serine and threonine residues in this motif determines the correct polarity of the *A. thaliana* PIN1 protein (Zhang *et al.*, 2010). Lack of motif 11 could play a role in the different polarities (up the gradient and with the flux) described for PIN1 and SoPIN1 proteins in the model proposed by O’Connor *et al.* (2014).

No free auxin could be detected in the pedicel samples analysed, probably due to the limits of detection of the method used. However, variations in levels of auxin precursors and conjugated auxin correlate with the observed changes in AtqPIN protein and AtqPIN1/SoPIN1 transcript levels and help to shed light on the changes in auxin metabolism during the process of bulbil formation. Before induction at time S0, levels of auxin precursors are highest whereas the auxin conjugate IAAsp is found at a relatively low concentration. The sharp drop in precursor levels following flower bud removal could reflect the response of plant tissue to synthesize and concentrate auxin locally, leading to the development of new meristems and vascular tissue, therefore consuming the precursor molecules, leading to a drop in concentration. As bulbil development progresses, auxin levels would increase to the S5 stage where the normal auxin localization patterns associated with apical meristem development are observed. The gradual rise in the level of the auxin conjugate could indicate that as auxin levels rise, more auxin is converted to the conjugate form either for storage or eventually to be destined for degradation. In regard to auxin levels, the initiation of bulbil formation may be compared with breaking of seed dormancy or the stimulation of axillary meristems since these processes also rely on a localized drop in auxin levels (Sorce *et al.*, 2009; He *et al.*, 2012; Liu *et al.*, 2013). The fluctuation in auxin levels may allow the activation by other plant hormones such as cytokinin or ABA of cells close to the bracteoles with the potential to form new meristems.

**Fig. 8.**
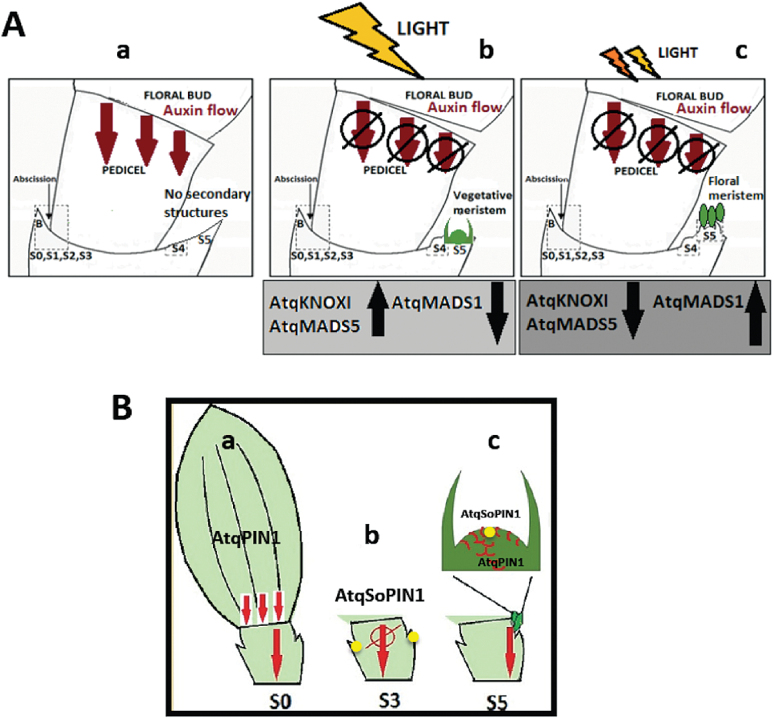
Model for the role of PIN1-mediated auxin flux in induction of secondary structures at the bracteoles in *A. tequilana* following removal of floral buds. (A) Auxin suppresses secondary structure formation. (a) A normal auxin gradient from the flower bud suppresses formation of secondary structures at the bracteoles. (b) Under natural light conditions, removal of flower buds leads to a drop in auxin flux in pedicel vascular tissue, stimulating the development of new meristems and vegetative bulbils at the bracteoles. (c) Under reduced light conditions, removal of flower buds leads to a drop in auxin flux in pedicel vascular tissue, stimulating the development of a mixture of determinate floral meristems producing non-viable floral structures and indeterminate meristems producing vegetative bulbils at the bracteoles. (B) Putative role of agave PIN1 proteins in bulbil formation. (a) Polarized AtqPIN1 is present in vascular tissue, leading to a downward auxin flux from the flower bud. (b) Removal of the flower bud interrupts the auxin flux. *AtqPIN1* expression decreases and *AtqSoPIN1* expression increases, allowing auxin to accumulate close to the bracteoles where new meristems will form (dots). (c) AtqSoPIN1 allows accumulation of auxin at the SAM of developing bulbil meristems and AtqPIN1 allows downward auxin flux and formation of vascular tissue. (This figure is available in colour at *JXB* online.)

Based on results in this and previous reports, a model for induction of bulbils in *A. tequilana* is proposed (Fig. 8). The model proposes that the basipetal auxin gradient from the flower bud suppresses formation of bulbils at the bracteoles. Under natural light conditions, removal of flower buds leads to a drop in auxin flux in pedicel vascular tissue, stimulating the development of new meristems and vegetative bulbils at the bracteoles. Under reduced light conditions, a mixture of determinate floral meristems producing non-viable floral structures and indeterminate meristems producing vegetative bulbils at the bracteoles develops. Putative roles for each agave PIN protein in bulbil formation can also be proposed as follows. Polarized AtqPIN1 is present in vascular tissue leading to a downward auxin flux from the flower bud. Removal of the flower bud interrupts this auxin flux and *AtqPIN1* expression decreases. In contrast, *AtqSoPIN1* expression increases, allowing auxin to accumulate close to the bracteoles where new meristems will form. In the final stage of bulbil induction, AtqSoPIN1 allows accumulation of auxin at the SAM of developing bulbil meristems, reinstating *AtqPIN1* expression and thus the downward auxin flux and formation of new vascular tissue.

## Supplementary data

Supplementary data are available at *JXB* online.

**Figure S1.** Stages of vegetative bulbil formation S0–S5 and corresponding histological images.

**Figure S2.** qRT-qPCR expression profiles of agave *KNOX and MADS* genes during the different stages of bulbil formation in pedicel tissue which produces floral structures at bracteoles.

**Figure S3.** Alignment of amino acid sequences of PIN1 proteins from *A. thaliana*, corn, rice sorghum, and agave.

**Figure S4.** Distribution within the variable hydrophilic domain of conserved amino acid motifs found in PIN1 and SoPIN1 proteins.

Table S1. Primers used for RT-qPCR.

Table S2. Levels of identity between complete amino acid sequences and hydrophilic domains of PIN1 and SoPIN1 proteins from maize and agave.

Table S3. Percentage identity of conserved amino acid motifs found in PIN1 and SoPIN1 proteins.

Table S4. Calibration parameters and sensitivity of the UHPLC-Q-TOF MS method.

Supplementary Data
