# Supplementary material for: Functionally different PIN proteins control auxin flux during bulbil development in Agave tequilana
Source: J Exp Bot. 2015 Apr 23;66(13):3893–905. doi: 10.1093/jxb/erv191 (PMC4473989; doi:10.1093/jxb/erv191)
Supplement: Supplementary Data [file supp_66_13_3893__index.html]

Functionally different PIN proteins control auxin flux during bulbil development in Agave tequilana — Supplementary Data 

# Functionally different PIN proteins control auxin flux during bulbil development in *Agave tequilana*

## Supplementary Data

Data files

**Files in this Data Supplement:**

- Supplementary Data - Supplementary Data
