# Supplementary material for: Functionally different PIN proteins control auxin flux during bulbil development in Agave tequilana
Source: J Exp Bot. 2015 Apr 23;66(13):3893–905. doi: 10.1093/jxb/erv191 (PMC4473989; doi:10.1093/jxb/erv191)
Supplement: Supplementary Data [file supp_erv191_erv191_Supplementaryapril2015.pdf]

Figure S1.

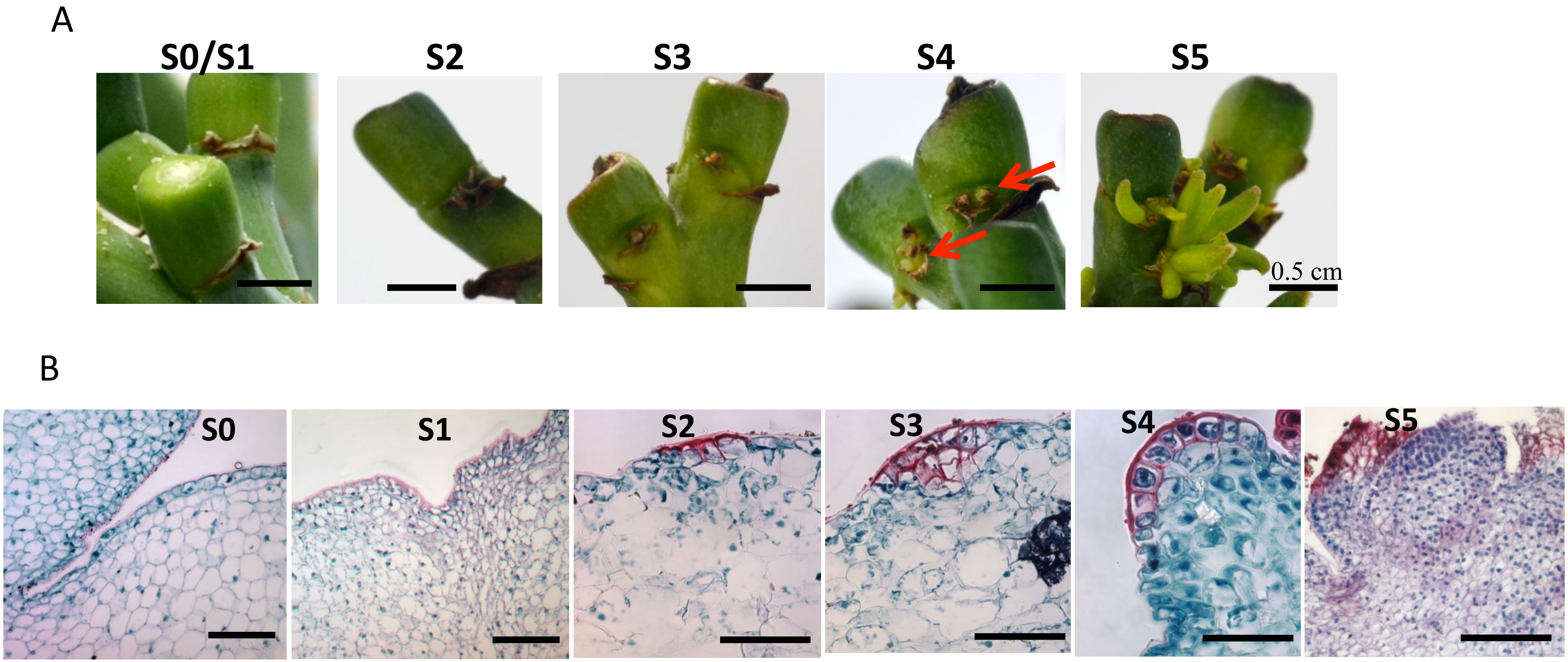

B: Histological analysis of samples in A

Figure S2.

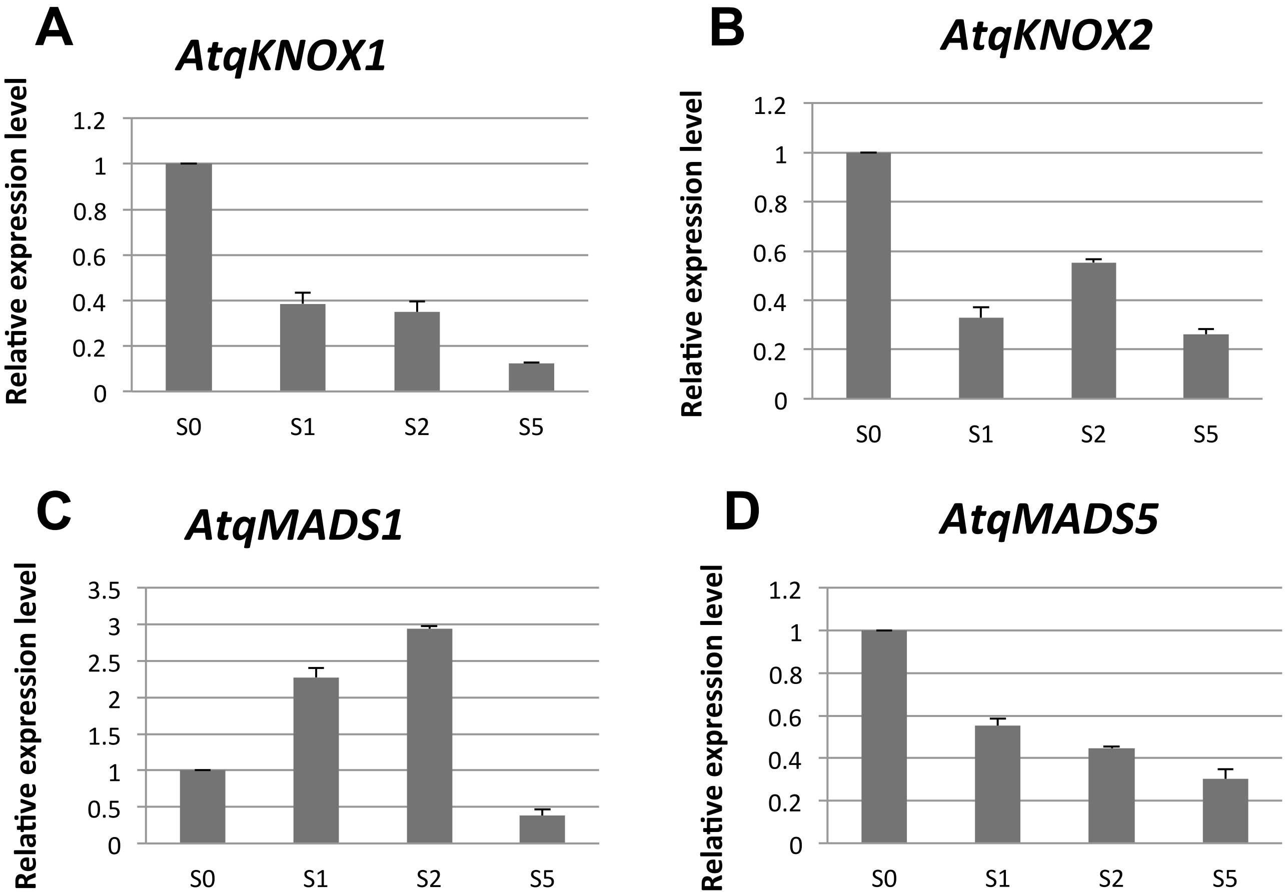

Figure S2. qRT-qPCR expression profiles of Agave *KNOX and MADS* genes during the different stages of bulbil formation in pedicel tissue which produces floral structures at bracteoles.

Figure S3.

```
AtqPIN1     1   MITLADFYHVMTAVVPLYVAMILAYGSVKWWKIFSPDQCSGINRFVALFAVPLLSFHFISTNNPYAMNMRFIAADTLQKLMVLALLTAWANLSRRG-----SLEWTITLFSLSTLPNTLVMGIPLLKGMYGDF--------SGSLMVQIV 137
AtPIN1      1   MITAADFYHVMTAMVPLYVAMILAYGSVKWWKIFTPDQCSGINRFVALFAVPLLSFHFIAANNPYAMNLRFLAADSLQKVIVLSLLFLWCKLSRNG-----SLDWTITLFSLSTLPNTLVMGIPLLKGMYGNF--------SGDLMVQIV 137
OsPIN1a     1   MITGADFYHVMTAMVPLYVAMILAYGSVKWWRIFTPDQCSGINRFVALFAVPLLSFHFISTNNPYTMNLRFIAADTLQKLIVLALLTLWSHLSRRG-----SLEWTITLFSLSTLPNTLVMGIPLLKGMYGEF--------SGSLMVQIV 137
OsPIN1b     1   MITAADFYHVMTAMVPLYVAMILAYGSVKWWRIFTPDQCSGINRFVALFAVPLLSFHFISTNNPYTMNLRFIAADTLQKLMVLAMLTAWSHLSRRG-----SLEWTITLFSLSTLPNTLVMGIPLLKGMYGEF--------SGSLMVQIV 137
ZmPIN1a     1   MITGTDFYHVMTAMVPLYVAMILAYGSVRWWRIFTPDQCSGINRFVALFAVPLLSFHFISTNNPYTMNIRFIAADTLQKLIVLALLTAWSYLSRRG-----CLEWTITLFSLSTLPNTLVMGIPLLKGMYGDF--------SGSLMVQIV 137
ZmPIN1b     1   MITGTDFYHVMTAVVPLYVAMILAYGSVRWWRIFSPDQCSGINRFVALFAVPLLSFHFISTNNPYTMNLRFIAADTLQKLMVLAMLTAWSHLSRRG-----SLEWTITLFSLSTLPNTLVMGIPLLKGMYGDF--------SGSLMVQIV 137
AtqSoPIN1   1   MISVSDLYHVLTAVVPLYVAMVLAYGSVKWWKIFTPDQCSGINRFVALFAVPLLSFHFISTNNPYAMNLRFIAADTLQKLLVLSLLSLWSWLASRG----CRLEWAITLFSLSTLPNTLVMGIPLLKGMYGDS--------SGSLMVQIV 138
ZmSoPIN1    1   MITALDLYHVLTAVVPLYVAMTLAYGSVRWWRIFTPDQCSGINRFVALFAVPLLSFHFISTNDPFAMNLRFLAADTLQKVAVLALLALASRGLSS--PRALGLDWSITLFSLSTLPNTLVMGIPLLRGMYG---ASS----AGTLMVQVV 141
SbPIN1      1   MITALDLYHVLTAVVPLYVAMTLAYGSVRWWRIFTPDQCSGINRFVALFAVPLLSFHFISTNNPFAMNLRFLAADTLQKVAVLALLFLASRLFSSSASRFLGLDWSITLFSLSTLPNTLVMGIPLLRGMYGGGGASSSSIDAGTLMVQVV 150
OsPIN1c     1   MITVVDLYHVLTAVVPLYVAMTLAYASVRWWRIFSPDQCSGINRFVALFAVPLLSFHFISTNNPFAMNLRFLAADTLQKLIVLALLALWCRLSARG-----SLDWLITLFSLSTLPNTLVMGIPLLKGMYAAA---ADVD-SGSLMVQIV 141
OsPIN1d     1   MITVVDLYHVLTAVVPLYVAMTLAYASVRWWRIFSPDQCSGINRFVALFAVPLLSFHFISTNNPFAMNLRFLAADTLQKLIVLALLALWCRLSARG-----SLDWLITLFSLSTLPNTLVMGIPLLKGMYAAAGAAAGAD-SGSLMVQIV 144

AtqPIN1     138 VLQCIIWYTLMLFMFEYRGAKLLITEQFPDT-AGSIASIAVDSDVMSLD---GRRDMLETEA-----------EIKEDGKLHVTVRRSNASRSD------IYSRRSQG-------FSTTTPRPSNLTNAEIYSLQSSRNPTPRGSSFNHT 259
AtPIN1      138 VLQCIIWYTLMLFLFEYRGAKLLISEQFPDT-AGSIVSIHVDSDIMSLD----GRQPLETEA-----------EIKEDGKLHVTVRRSNASRSD------IYSRRSQG--------LSATPRPSNLTNAEIYSLQSSRNPTPRGSSFNHT 257
OsPIN1a     138 VLQCIIWYTLMLFMFEYRGARILITEQFPDT-AGAIASIVVDADVVSLD---GRRDMIETEA-----------EVKEDGKIHVTVRRSNASRSD------VYSRRSMG-------FSSTTPRPSNLTNAEIYSLQSSRNPTPRGSSFNHT 259
OsPIN1b     138 VLQCIIWYTLMLFMFEYRGARMLITEQFPDT-AANIASIVVDPDVVSLD---GRRDAIETET-----------EVKEDGRIHVTVRRSNASRSD------IYSRRSMG-------FSSTTPRPSNLTNAEIYSLQSSRNPTPRGSSFNHT 259
ZmPIN1a     138 VLQCIIWYTLMLFMFEYRGARILITEQFPDT-AGAIASIVVDPDVVSLD---GRNDAIETEA-----------EVKEDGKIHVTVRRSNASRSD------IYSRRSMG-------FSSTTPRPSNLTNAEIYSLQSSRNPTPRGSSFNHT 259
ZmPIN1b     138 VLQCIIWYTLMLFMFEYRGARMLITEQFPDN-AGAIASIVVDPDVVSLD---GRRDAIETEA-----------EVKEDGRIHVTVRRSNASRSD------IYSRRSMG-------FSSTTPRPSNLTNAEIYSLQSSRNPTPRGSSFNHN 259
AtqSoPIN1   139 VLQCIIWYTLMLFLFEYRGAKLLIMEQFPD-TGASIVSFRVDSDILSLDGK----EPLQTDA-----------ELGEDGKLHVTVRKSTSSSRDRSDIFSGRSHGHHSGGLGLGGMSSVTPRASNLTNAEIYSLQSSRNPTPRGSSFNHT 272
ZmSoPIN1    142 VLQCIIWYTLMLFLFEYRAARALVLDQFPDGAAASIVSFRVDSDVVSLARG---DVELEAEPDGVAGAGAVSSRGGDAGRVRVTVRKSTSSRSE-----AACSH-SHS--------QTMQPRVSNLSGVEIYSLQSSRNPTPRGSSFNHA 274
SbPIN1      151 VLQCIIWYTLMLFLFEYRAARALVLDQFPDGAAASIVSFRVDSDVVSLARG---EIELEADAVPVP-------AGGDGGRVRVTVRKSTSSRSE-----AACSH-SHSH------SQSMQPRVSNLSGVEIYSLQSSRNPTPRGSSFNHT 278
OsPIN1c     142 VLQCIIWYTLMLFLFEYRGARLLVMEQFPD-TAASIVSFRVDSDVVSLAGGGGGAAELQAEA-----------EVGDDGRMRVTVRKSTSSRSE-----AACSHGTQSH------SQSMQPRVSNLSGVEIYSLQSSRNPTPRGSSFNHA 268
OsPIN1d     145 VLQCIIWYTLMLFLFEYRGARLLVMEQFPD-TAASIVSFRVDSDVVSLAGGGGGAAELQAEA-----------EVGDDGKMRVTVRKSTSSRSE-----AACSHGTQSH------SQSMQPRVSNLSGVEIYSLQSSRNPTPRGSSFNHA 271

AtqPIN1     260 DFYS---MVG----RSSNFGAS-DAFGVVAAGVPRGPNTPRPSNYEEEHGGAAAPAAAAAANPAAAAGAGKPRYHYQMPANGGTAHYPAPNPCVFSPAGPTPLAPKAAVASARKTANGQAHLL--KPEEGGVTKDLHMYVWSSSASPVS- 398
AtPIN1      258 DFYS---MMASGGGRNSNFGPGEAVFG--SKGP-----TPRPSNYEEDGGPAKPTAAGTAAGAGRFHYQSGG------SGGGGGAHYPAPNPGMFSPN---------TGGGGGTAAKGNAPVVGGKRQDGN-GRDLHMFVWSSSASPVS- 380
OsPIN1a     260 DFYS---MVG----RSSNFAAG-DAFG-VRTGA-----TPRPSNYEEDAAAPNKAGSKY-------------------------GQYPAPNPAMAAPP----------KP--KKAANGQA-----KGEDG---KDLHMFVWSSSASPVS- 349
OsPIN1b     260 DFYS---MVG----RSSNFGAA-DAFG-VRTGA-----TPRPSNYEDDASKPKYPLPASNAAPMA-------------------GHYPAPNPAVSSAP----------KGAKKAATNGQA-----KGED------LHMFVWSSSASPVS- 354
ZmPIN1a     260 DFYS---MVG----RSSNFAAG-DAFG-LRTGA-----TPRPSNYEED---PQGKANKY-------------------------GQYPAPNPAMAAQP----------AKGLKKAANGQA-----KGEDG---KDLHMFVWSSSASPVS- 348
ZmPIN1b     260 DFYS---MVG----RSSNFGAA-DAFG-IRTGA-----TPRPSNYEDDASKPKYPLPVVNATSGAGA-----------------AHYPAPNPAVAAAP----------KGARKAATNGQA-----KGED------LHMFVWSSSASPVS- 356
AtqSoPIN1   273 DTDFVNG------GRSSNFDFGGNASQLLDDLER-----ANCANVSNSNGLFAAPAAGKK---------------------------ENNN------------------VLNGSREGGKD---------------LHMFVWSSSASPVSE 351
ZmSoPIN1    275 D----------------FFNIVGAAAKGGGG-----------AAGDEEKGACGGGGGGHS---------------------------PQPQ------------------AVA-VPAKRKD---------------LHMLVWSSSASPVSE 336
SbPIN1      279 D----------------FFNIVGAGAKGGAAAA---------AAGDEEK----GGAGGHS---------------------------PQPQ------------------AVAAVAAKRKD---------------LHMLVWSSSASPVSE 339
OsPIN1c     269 E----------------FFNIVGNGKQ-----------------GDEEKG--AAGGGGHS---------------------------PQP-----------------------VVGKRKD---------------LHMFVWSSSASPVSE 318
OsPIN1d     272 E----------------FFNIVGNGKH-----------------GDEEKG--AAGGGGHS---------------------------PQP-----------------------VVGKRKD---------------LHMFVWSSSASPVSE 321

AtqPIN1     398 -----------DVFGNN-QEYALHAAADPAANAAVKDVRLAVSPGKVKENRDDYMAERDEFSFGNRGMMDRDGDHDGGDEKMHEPTT------KVGRATAMPPTSVMTRLILIMVWRKLIRNPNTYSSLIGLTWSLVSFKWHVQMPAIIS 530
AtPIN1      380 -----------DVFGGGGGNHHADYSTATN-----DHQKDVKISVPQGNSNDNQYVEREEFSFGNK-------DDDSKVLATDGGNNISN---KTTQAKVMPPTSVMTRLILIMVWRKLIRNPNSYSSLFGITWSLISFKWNIEMPALIA 504
OsPIN1a     349 -----------DVFGNG-AEYN-DAAAVKEVRMAVASPRK--ADG----------VERDDFSFGNRGVAERDAEAGDEKSVAAAVSGEHGKPGLTPAPTAMPPTSVMTRLILIMVWRKLIRNPNTYSSLIGLIWSLVCFRWNFEMPAIIL 474
OsPIN1b     354 -----------DVFGGGAPDYN-DAAAVK-------SPRK--MDGAK-DRED--YVERDDFSFGNRGVMDRDAEAGDEKAAAAAGADPSK---AMAAPTAMPPTSVMTRLILIMVWRKLIRNPNTYSSLIGLIWSLVCFRWNFEMPAIVL 477
ZmPIN1a     348 -----------DVFGNGAAEYN-DAAAVKEVRMAVASPRKVAADGRKERGED--FAERDDFSFGNRGAAERDAEAGDEK-AAAAAAAAQGNAGAVAAPAAMPPTSVMTRLILIMVWRKLIRNPNTYSSLIGLIWSLVCFRWNFEMPAIIL 483
ZmPIN1b     356 -----------DVFGGGAPDYN-EAAAAK-------SPRK--MDGGK-DRDD--YVERDEFSFGNRGAMDRDAEAGDG-KAAAAGGDPS----TVAAPTAMPPTSVMTRLILIMVWRKLIRNPNTYSSLIGVIWSLVCFRWNFQMPAIVL 477
AtqSoPIN1   352 GGG-------LPVFRGGLAEFGK----EKINT----AEYGRDEFSFRSRPMALGVVEDAAMSMQRDNNIGRSNSTAELRPKASDCGDGDL----KP-AAAMPPASVMTRLILIMVWRKLIRNPNTYSSLLGLIWSLVSFRWGIVMPAIIA 481
ZmSoPIN1    337 RAA-------VHVFGAGGADHADVLAKGAQAY----DEYGRDDYSSRTK-NGSGGADKGGPTLS---KLG-SNSTAQLYPKD----DGEG----RAAAVAMPPASVMTRLILIMVWRKLIRNPNTYSSLIGVVWSLVSYRWGIEMPAIIA 462
SbPIN1      340 RAAG----AAVHVFGAGAADHADVLAKGTQAY----DEYGRDDFS-RTK-NGNG-ADKGGPTLS---KLG-SNSTAQLYPKD----DGEG----RP-AAAMPPASVMTRLILIMVWRKLIRNPNTYSSLIGVIWSLVSYRWGIQMPAIIA 465
OsPIN1c     319 RAAAAAAG-AVHVFGGGGADHGD--AKGAQAY----DEYSFGNKN-----------EKDGPTLS---KLG-SNSTAQLRPKD----DGEG----MA--AAMPPASVMTRLILIMVWRKLIRNPNTYSSLLGVIWSLVSYRWGIEMPAIIA 436
OsPIN1d     322 RAAAAAAAGAVHVFGGGGADHGD--AKGAQAY----DEYSFGNKN-----------EKDGPTLS---KLG-SNSTAQLRPKD----DGEG----RA--AAMPPASVMTRLILIMVWRKLIRNPNTYSSLLGVIWSLVSYRWGIEMPAIIA 440

AtqPIN1     531 KSISILSDAGLGMAMFSLGLFMALQPRIIACGNRVAGFSMAVRFLAGPAVMAAASIAVGLRGALLHVAIVQAALPQGIVPFVFSKEYNVHPDILSTGVIFGMLIALPITLVYYIVLGL 648
AtPIN1      505 KSISILSDAGLGMAMFSLGLFMALNPRIIACGNRRAAFAAAMRFVVGPAVMLVASYAVGLRGVLLHVAIIQAALPQGIVPFVFAKEYNVHPDILSTAVIFGMLIALPITLLYYILLGL 622
OsPIN1a     475 KSISILSDAGLGMAMFSLGLFMALQPRIIACGNKVATFAMAVRFLTGPAVMAAASIAVGLRGTLLHVAIVQAALPQGIVPFVFAKEYSVHPDILSTAVIFGMLIALPITLVYYILLGL 592
OsPIN1b     478 KSISILSDAGLGMAMFSLGLFMALQPHIIACGNKVATYAMAVRFLAGPAVMAAASFAVGLRGTLLHVAIVQAALPQGIVPFVFAKEYSVHPSILSTAVIFGMLIALPITLVYYILLGL 595
ZmPIN1a     484 KSISILSDAGLGMAMFSLGLFMALQPRIIACGNKVATFAMAVRFLTGPAVMAAASFAVGLRGTLLHVAIVQAALPQGIVPFVFAKEYGVHPDILSTAVIFGMLIALPITLVYYILMGL 601
ZmPIN1b     478 QSISILSDAGLGMAMFSLGLFMALQPRIIACGNKVATFAMAVRFLTGPAVMAAASFAVGLRGTLLHVAIVQAALPQGIVPFVFAKEYNVHPDILSTAVIFGMLIALPITLVYYILLGL 595
AtqSoPIN1   482 RSIAILSDAGLGMAMFSLGLFMALQPRIIACGNTIAAFAMGVRFITGPAVMAVASIAVGLRGVLLHIAIVQAALPQGIVPFVFAKEYNVHPDILSTGVIFGMLIALPITLVYYILLGL 599
ZmSoPIN1    463 RSISILSDAGLGMAMFSLGLFMALQPRIIACGNKLAAIAMGVRFVAGPAVMAAASIAVGLRGVLLHIAIVQAALPQGIVPFVFAKEYGVHPDILSTAVIFGMLIALPITLVYYILLGL 580
SbPIN1      466 RSISILSDAGLGMAMFSLGLFMALQPRIIACGNKFAVIAMAVRFLAGPAVMAAASIAVGLRGVLLHIAIVQAALPQGIVPFVFAKEYNVHPDILSTAVIFGMLIALPITLVYYILLGL 583
OsPIN1c     437 RSISILSDAGLGMAMFSLGLFMALQPRIIACGNSLASYAMAVRFLVGPAVMAAASIAVGLRGVLLHIAIVQAALPQGIVPFVFAKEYNVHPNILSTAVIFGMLIALPITLVYYILLGL 554
OsPIN1d     441 RSISILSDAGLGMAMFSLGLFMALQPRIIACGNSLASYAMAVRFLVGPAVMAAASIAVGLRGVLLHIAIVQAALPQGIVPFVFAKEYNVHPNILSTAVIFGMLIALPITLVYYILLGL 558
```

Figure S3. Alignment of amino acid sequences of PIN1 proteins from *A. thaliana*, corn, rice sorghum and agave. Transmembrane domains are underlined and the antigenic region used to produce the Anti-ZmPIN1a antibody is highlighted in pink. Atq-*A. tequilana*, At-*A. thaliana*, Os-*O. Sativa*, Zm-*Z. mays*, Sb-*S. bicolor*, So-Sister of PIN

Figure S4.

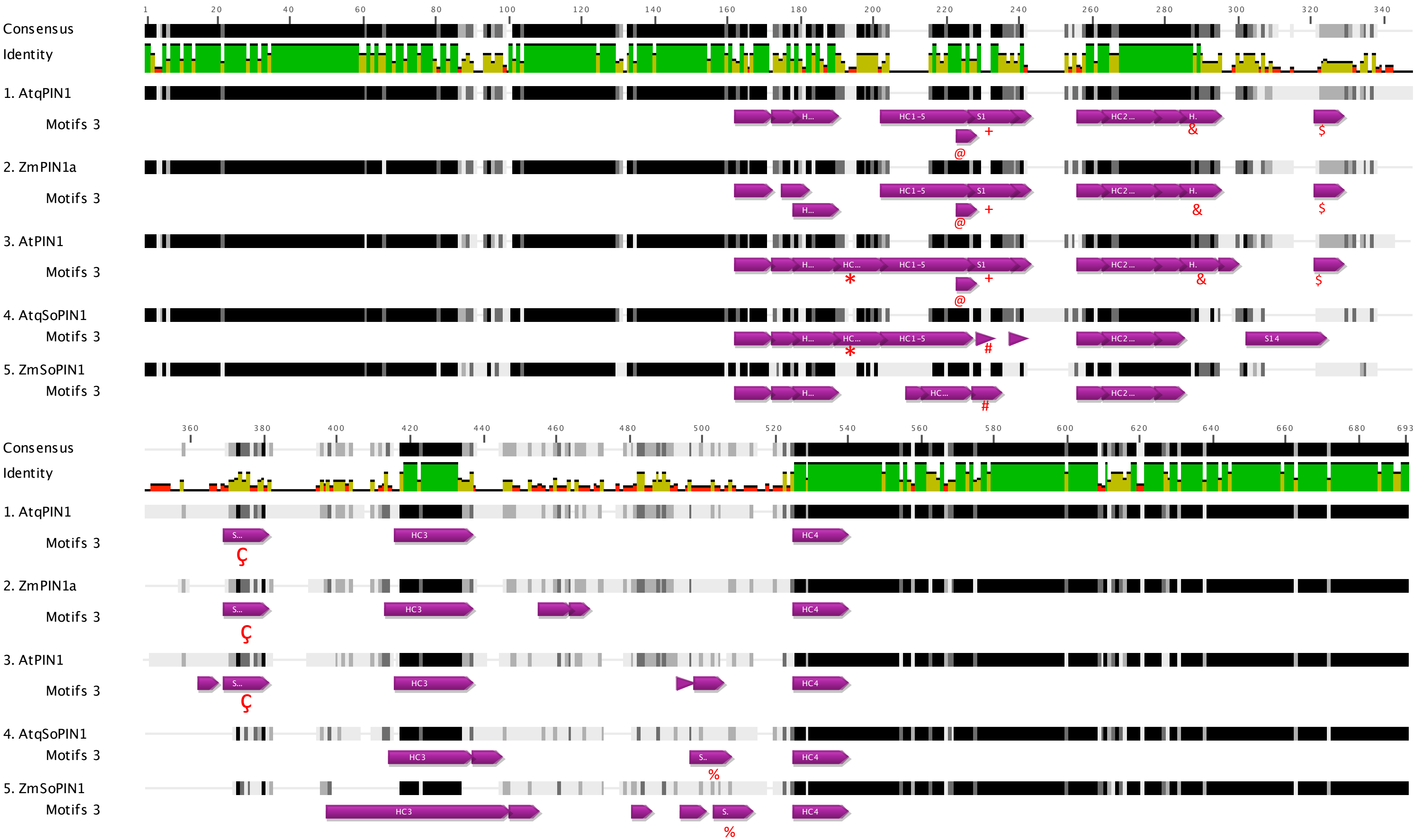
Consensus
Identity
1. AtqPIN1
Motifs 3
2. ZmPIN1a
Motifs 3
3. AtPIN1
Motifs 3
4. AtqSoPIN1
Motifs 3
5. ZmSoPIN1
Motifs 3
HC1-5
S1
HC2
HC3
HC4
S14

Figure S4. Distribution within the variable hydrophylic domain of conserved amino acid motifs found in PIN1 and SoPIN1 proteins. *-HC1-4 motif, @-S2 motif, +-S1 motif, #-S7 motif, &-HC2-4 motif, $-HC2-1-1 motif, ç-S11 motif, %-S20 motif. Some repeated motifs have been removed for clarity.

Table S1. Primers used for RT-qPCR

Specific primer	Sequence
AtqPIN1-F	5´-GTCCCATTCGTGTTCTCAAAGG-3
AtqPIN1-R	5´-TGCAATCAGCATCCCGAATA-3
AtqSoPIN1-F	5´-GGGCTAACTGCGCTAATGTCA-3
AtqSoPIN1-R	5´-CCCTTCTCGTGATCCATTCAG-3
AtqACT2-F	5´-GTACAACCGGTATTGTGCTGGAT-3
AtqACT2-R	5´-GCTTGGTGCAAGTGCTGTGATTTC-3

Table S2. Levels of identity between complete amino acid sequences and hydrophilic domains of PIN1 and SoPIN1 proteins from maize and agave

Complete sequences

**%Identities**	AtqPIN1	AtqSoPIN1	ZmPIN1a	ZmSoPIN1
AtqPIN1	100			
AtqSoPIN1	57.3	100		
ZmPIN1a	**72.1**	60.3	100	
ZmSoPIN1	52.6	**62.4**	56.2	100

Hydrophilic domain sequences

**%Identities**	AtqPIN1	AtqSoPIN1	ZmPIN1a	ZmSoPIN1
AtqPIN1	100			
AtqSoPIN1	15.5	100		
ZmPIN1a	**38.1**	19.8	100	
ZmSoPIN1	14.4	**28.4**	16.0	100

Table S3. Percentage identity of conserved amino acid motifs found in PIN 1 and SoPIN1 proteins

PIN protein	Name	% Identity	Motif
AtPIN1	S11	63.6	GYPAPNPGFSP
AtPIN1	HC1-1	88.9	LLIMEQFPD
AtPIN1	HC1-2	83.3	TAASIV
AtPIN1	HC1-3	63.6	SFKVDSDVVSL
AtPIN1	S14	57.1	LHVFGGN
AtPIN1	HC1-4	66.7	DGREPVQTEAEV
AtPIN1	HC1-5	84.6	GEDGKLHVTVRKS
AtPIN1	S2	75	RRSQ
AtPIN1	S2	100	RRSQ
AtPIN1	S1	55.6	VSSRSQISS
AtPIN1	HC3	70	AKELHMFVWSSSASPVSEAG
AtPIN1	HC4	78.6	MPPSAVMTRLILDM
AtPIN1	S21	75	DDAK
AtPIN1	HC2-2	92.9	LTGAEIYSLQSSRN
AtPIN1	HC2-3	71.4	LTPRESS
AtPIN1	S4	75	SGRG
AtPIN1	S4	75	SGRG
AtPIN1	HC2-4	90	FMHTDFYSMM
AtPIN1	HC2-1	85.7	LTPRPSN
AtPIN1	HC2-1-1	85.7	LTPRPSN

PIN protein	Name	% Identity	Motif
AtqPIN1	S11	54.5	GYPAPNPGFSP
AtqPIN1	HC1-1	88.9	LLIMEQFPD
AtqPIN1	HC1-2	66.7	TAASIV
AtqPIN1	HC1-3	72.7	SFKVDSDVVSL
AtqPIN1	HC1-5	84.6	GEDGKLHVTVRKS
AtqPIN1	S2	75	RRSQ
AtqPIN1	S2	100	RRSQ
AtqPIN1	S1	55.6	VSSRSQISS
AtqPIN1	HC3	70	AKELHMFVWSSSASPVSEAG
AtqPIN1	HC4	78.6	MPPSAVMTRLILDM
AtqPIN1	HC2-2	92.9	LTGAEIYSLQSSRN
AtqPIN1	HC2-3	71.4	LTPRESS
AtqPIN1	HC2-4	80	FMHTDFYSMM
AtqPIN1	HC2-1	85.7	LTPRPSN
AtqPIN1	HC2-1-1	85.7	LTPRPSN

PIN protein	Name	% Identity	Motif
AtqSoPIN1	HC1-1	100	LLIMEQFPD
AtqSoPIN1	HC1-2	83.3	TAASIV
AtqSoPIN1	HC1-3	72.7	SFKVDSDVVSL
AtqSoPIN1	S14	57.1	LHVFGGN
AtqSoPIN1	S14	57.1	LHVFGGN
AtqSoPIN1	HC1-4	66.7	DGREPVQTEAEV
AtqSoPIN1	HC1-5	100	GEDGKLHVTVRKS
AtqSoPIN1	HC3	85	AKELHMFVWSSSASPVSEAG
AtqSoPIN1	HC4	78.6	MPPSAVMTRLILDM
AtqSoPIN1	S20	80	SSSTAELHPK
AtqSoPIN1	HC2-2	92.9	LTGAEIYSLQSSRN
AtqSoPIN1	HC2-3	71.4	LTPRESS
AtqSoPIN1	S4	75	SGRG
AtqSoPIN1	S7	75	SSRG
AtqSoPIN1	HC2-1	71.4	LTPRPSN

PIN protein	Name	% Identity	Motif
ZmPIN1a	S10	75	SPRF
ZmPIN1a	S11	54.5	GYPAPNPGFSP
ZmPIN1a	HC1-1	77.8	LLIMEQFPD
ZmPIN1a	HC1-2	66.7	TAASIV
ZmPIN1a	HC1-3	72.7	SFKVDSDVVSL
ZmPIN1a	S15	75	AKEVRMLV
ZmPIN1a	HC1-5	76.9	GEDGKLHVTVRKS
ZmPIN1a	S2	75	RRSQ
ZmPIN1a	S2	75	RRSQ
ZmPIN1a	S1	55.6	VSSRSQISS
ZmPIN1a	HC3	75	AKELHMFVWSSSASPVSEAG
ZmPIN1a	HC4	78.6	MPPSAVMTRLILDM
ZmPIN1a	HC2-2	92.9	LTGAEIYSLQSSRN
ZmPIN1a	HC2-3	71.4	LTPRESS
ZmPIN1a	HC2-4	80	FMHTDFYSMM
ZmPIN1a	HC2-1	85.7	LTPRPSN
ZmPIN1a	HC2-1-1	85.7	LTPRPSN

PIN protein	Name	% Identity	Motif
ZmSoPIN1	HC1-1	55.6	LLIMEQFPD
ZmSoPIN1	HC1-2	83.3	TAASIV
ZmSoPIN1	HC1-3	90.9	SFKVDSDVVSL
ZmSoPIN1	S14	57.1	LHVFGGN
ZmSoPIN1	HC1-5	61.5	GEDGKLHVTVRKS
ZmSoPIN1	S18	66.7	DGPSLS
ZmSoPIN1	HC3	75	AKELHMFVWSSSASPVSEAG
ZmSoPIN1	HC4	78.6	MPPSAVMTRLILDM
ZmSoPIN1	S20	70	SSSTAELHPK
ZmSoPIN1	HC2-2	85.7	LTGAEIYSLQSSRN
ZmSoPIN1	HC2-3	71.4	LTPRESS
ZmSoPIN1	S7	100	SSRG
ZmSoPIN1	S7	75	SSRG
ZmSoPIN1	S7	75	SSRG
ZmSoPIN1	HC2-1	57.1	LTPRPSN

Table S4. Calibration parameters and sensitivity of the UHPLC-Q-TOF MS method.

Metabolite	RT min	RT RSD%	Calibration range ng/mL	Correlation coefficient (R2)	LOD ng/mL	LOQ ng/mL	Extraction %Recovery
Triptophan (TRP)	2.6	0.28	10-600	0.9998	1.51	5.04	41.58
Tryptamine (TRA)	1.4	0.82	10-600	0.9992	0.35	1.15	43.96
Indole-3-acetic acid (IAA)	8.3	0.11	10-600	0.9980	2.85	9.49	70.99
Indole-3-acetyl-l-aspartic acid (IAAsp)	6.9	0.20	10-600	0.9980	4.94	16.47	94.79
